# Meal Timing, Aging, and Metabolic Health

**DOI:** 10.3390/ijms20081911

**Published:** 2019-04-18

**Authors:** Katharina Kessler, Olga Pivovarova-Ramich

**Affiliations:** 1 Research Group Molecular Nutritional Medicine, Department of Molecular Toxicology, German Institute of Human Nutrition Potsdam-Rehbruecke, 14558 Nuthetal, Germany; olga.ramich@dife.de; 2 German Center for Diabetes Research (DZD), 85764 München-Neuherberg, Germany; 3 Department of Endocrinology, Diabetes and Nutrition, Campus Benjamin Franklin, Charité University of Medicine, 12203 Berlin, Germany; 4 Department of Veterinary Medicine, University of Cambridge, Cambridge CB3 0ES, UK

**Keywords:** circadian clock, meal timing, chrononutrition, metabolic health, aging

## Abstract

A growing body of evidence suggests that meal timing is an important factor for metabolic regulation and that the circadian clock tightly interacts with metabolic functions. The proper functioning of the circadian clock is critical for maintaining metabolic health. Therefore, chrononutrition, a novel discipline which investigates the relation between circadian rhythms, nutrition, and metabolism, has attracted increasing attention in recent years. Circadian rhythms are strongly affected by obesity, type 2 diabetes, and other dietary-induced metabolic diseases. With increasing age, the circadian system also undergoes significant changes which contribute to the dysregulation of metabolic rhythms. Metabolic diseases are a major health concern, particularly in light of a growing aging population, and effective approaches for their prevention and treatment are urgently needed. Recently, animal studies have impressively shown beneficial effects of several dietary patterns (e.g., caloric restriction or time-restricted feeding) on circadian rhythms and metabolic outcomes upon nutritional challenges. Whether these dietary patterns show the same beneficial effects in humans is, however, less well studied. As indicated by recent studies, dietary approaches might represent a promising, attractive, and easy-to-adapt strategy for the prevention and therapy of circadian and metabolic disturbances in humans of different age.

## 1. Introduction

In humans, as in other living organisms, behavior and physiology are regulated by the circadian clock, which permits adaptation to the dramatically different phases of the day resulting from the Earth’s rotation. In particular, human metabolism is increasingly recognized as being highly regulated by circadian rhythms which are approximately 24 h long (“circa diem”). Circadian rhythms separate incompatible biochemical and physiological processes, optimize energy expenditure, and synchronize metabolic pathways [1]. Thus, proper functioning of circadian clocks is critical for maintaining metabolic health. Circadian disruption, e.g., by chronical shift work, leads to dysregulation of metabolic homeostasis and is associated with increased risk of obesity, metabolic syndrome, and cardiovascular diseases [2,3,4]. Circadian rhythms, in turn, are strongly affected by obesity, type 2 diabetes, and other metabolic diseases [5,6,7]. With advancing age, the circadian system undergoes significant changes, a process which is considered to contribute to the dysregulation of metabolic rhythms and development of aging-associated metabolic pathologies [8]. Metabolic diseases are a major health concern, in particular in light of a growing aging population [9], and effective approaches for their prevention and treatment are urgently needed.

Centuries ago, a medieval philosopher, Maimonides (1135–1204), coined the saying “Eat breakfast like a king, lunch like a prince and dine like a pauper”. At present, a growing body of evidence suggests that meal timing strongly contributes to the regulation of metabolic state and body weight [10,11,12]. It appears that meal time-based strategies can be employed to prevent obesity and associated metabolic diseases in young and older individuals. Chrononutrition, a novel discipline which investigates the relation between circadian rhythms, nutrition, and metabolism, has therefore attracted increasing attention in recent years [13,14]. The word “chrononutrition” means that meal timing is coordinated with the body’s circadian rhythms and reflects the idea that the timing of food intake is as critical as the food quantity and quality.

In this review, we summarize the current knowledge about the role of meal timing in metabolic regulation. We start with an overview of key molecular mechanisms of the circadian clock and clock-dependent metabolic regulation. Further, we describe disturbances of circadian rhythms associated with metabolic diseases and increasing age. We then summarize animal and human studies suggesting that meal timing is an important factor for metabolic regulation and describe underlying physiological and molecular mechanisms. We present data indicating that some meal timing-based dietary strategies improve circadian rhythms and clock-controlled metabolic functions. In this paper, we focus on time-restricted feeding and only touch on other dietary approaches. Finally, we discuss future studies which are needed to understand the effects of meal timing-based strategies and discuss their potential clinical impact for the prevention and treatment of metabolic diseases.

## 2. Circadian Regulation of Metabolism

**Figure 1:**
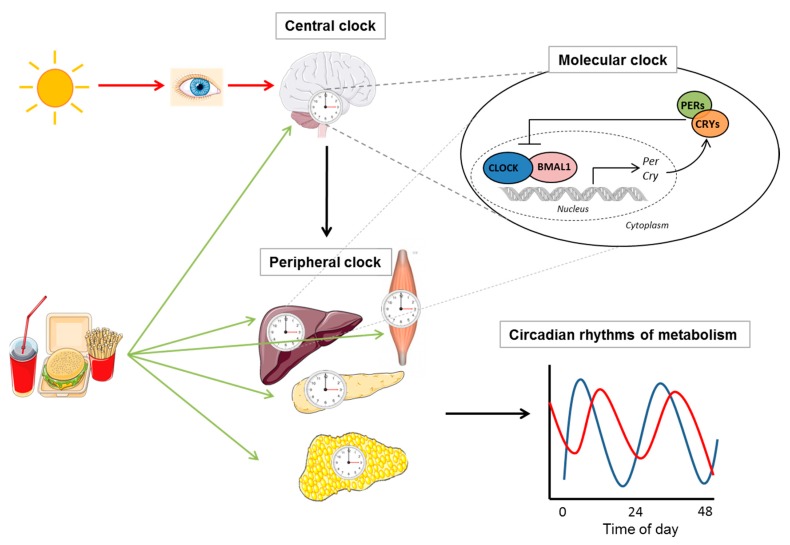
Circadian regulation of metabolism. In mammals, the circadian clock consists of a master clock in the suprachiasmatic nucleus (SCN) of the hypothalamus, which is synchronized by light/dark signals, and peripheral clocks, which is orchestrated by the master clock and controls metabolic rhythms. Food consumption can also entrain the endogenous clock but has a stronger influence on peripheral clocks than on the SCN. Illustrations, used in this Figure, were adapted from Servier Medical Art (http://smart.servier.com/). Servier Medical Art by Servier is licensed under a Creative Commons Attribution 3.0 Unported License.

In mammals, the circadian clock consists of a master clock in the suprachiasmatic nucleus (SCN) of the hypothalamus, which is synchronized by light/dark signals, and a peripheral clock orchestrated by a master clock via the nervous and humoral pathways [1]. Peripheral clock oscillators are found in almost all tissues, including in the liver, heart, kidney, intestine, skeletal muscles, and adipocytes and in peripheral blood cells. Food consumption represents an external input (“*Zeitgeber*”) which entrains the circadian clock, affecting primarily the peripheral circadian clocks (and, to a lesser extent, the central clock) (Figure 1). Thus, daily rhythms of metabolism arise from a complex interplay of the endogenous autonomous clock, exposure to light/darkness, and patterns of fasting/feeding.

The molecular mechanism of the circadian clock, which exists in almost every cell of our body, consists of interlocked transcriptional–translational feedback loops [11]. In the first loop, a heterodimer of the transcription factors aryl hydrocarbon receptor nuclear translocator like (ARNTL, also known as BMAL1) and clock circadian regulator (CLOCK) or neuronal PAS domain protein 2 (NPAS2) activates the transcription of genes *period (PER)*, *cryptochrome (CRY)*, *retinoic acid-related orphan receptors (RORs)* and *nuclear receptor subfamily 1 group D (NR1D1/2*, also known as *Rev-Erb*α/ß) by binding to E-box elements in their promoter regions. The resulting PER and CRY proteins heterodimerize, translocate to the nucleus, and interact with the BMAL1/CLOCK complex to inhibit their own transcription. In the second loop, ROR activates and REV-ERB represses RORE-mediated transcription of *BMAL1* and *CLOCK*. Numerous posttranslational modifications of circadian clock proteins such as phosphorylation, acetylation, ribosylation, SUMOylation and ubiquitination fine-tune the circadian function. Moreover, clock proteins interact with a number of coactivators, corepressors, and chromatin-associated factors in the regulation of target genes.

The clock mechanism controls rhythmic expression of numerous genes, i.e., clock-controlled genes (CCG), which are mostly transcription factors or rate limiting enzymes, which in turn arrange rhythms of metabolic genes and processes. Numerous molecular links between the core clock and metabolic pathways include REV-ERBs, RORs, peroxisome proliferator-activated receptors (PPARs), peroxisome proliferator-activated receptor gamma coactivator 1-alpha (PGC-1α), AMP-activated protein kinase (AMPK), sirtuin 1 (SIRT1), mammalian target of rapamycin (mTOR) and are described in detail in a range of excellent reviews [1,11,15]. Therefore, a large part of the transcriptome, proteome and metabolome displays circadian oscillations including components of carbohydrate, cholesterol, lipid, and energy metabolism, detoxification pathways, and inflammatory responses [16,17,18]. Genetic clock disruption leads to dysfunctions of glucose and lipid metabolism and development of obesity, type 2 diabetes, and associated metabolic diseases. Studies in mice with knockouts in core clock genes showed that different clock genes contribute to the regulation of different metabolic processes and that this regulation is tissue-specific [19,20,21]. 

In turn, the circadian clock itself undergoes metabolic regulation. In a healthy state, cyclical expression of metabolic regulators coordinates cellular processes for efficient metabolism. By contrast, metabolic disturbances induced by nutrient imbalance or excess result in blunted circadian regulation of metabolic pathways [22]. In mouse experiments, a high-fat diet (HFD) induced a dysregulation of the rhythms of locomotor and feeding behavior, reduction of the oscillation amplitude of core clock genes, and a profound reorganization of entire circadian transcriptome and metabolome, as well as an alteration of circadian rhythms of metabolic hormones (e.g., of insulin and leptin) [17,23,24]. Mouse models of obesity and diabetes also demonstrated an altered expression pattern of clock genes in liver, adipose tissue, heart, and other organs and an altered feeding rhythm [25,26]. Notably, in mice, HFD-induced transcriptional and epigenetic changes of the circadian clock arise before the onset of obesity and are reversible [17]. Mice fed an HFD for 10 weeks followed by 2 weeks of normal chow feeding remained significantly overweight relative to normal chow-fed littermates but showed restored circadian expression and chromatin recruitment of core clock genes [17]. We recently demonstrated that, in healthy humans, an isocaloric HFD increased the expression levels and amplitudes of core clock genes in blood monocytes, thereby affecting their diurnal oscillation, and delayed the 24-h salivary cortisol rhythm, which was used as a central clock marker [27]. A blunted rhythm of clock gene expression in blood leucocytes was also found in subjects with type 2 diabetes [5]. Altered clock gene expression in human adipose tissue was associated with obesity and metabolic syndrome [6,7]. Thus, dietary induced circadian clock disruption might be one of the factors contributing to the pathogenesis of metabolic disturbances.

## 3. Circadian Rhythms in Later Life

During the last decade, a range of studies has shown interconnections between the circadian clock and aging. Aging induces dysfunction of multiple physiological processes and is considered a separate risk factor for the development of diabetes, cancer, neurodegeneration, and sarcopenia [9]. Notably, circadian clock disruption was shown to contribute to the development of these pathologies [28]. Further, genetic disruption of the circadian clock, experimental jet lag or artificially short or long light/dark cycles result in a reduced lifespan accompanied by metabolic disturbances [29,30,31]. By contrast, a murine model of longevity (α-MUPA mice) showed high amplitude of the clock gene expression rhythms in the liver, and they live longer compared to wild type mice [32]. Effects of the circadian clock on longevity and aging-associated disease could be explained by the involvement of the circadian clock in the regulation of oxidative stress, cell cycle, cell death, proteolysis, and DNA damage response [33]. Thus, dysregulation of the circadian clock might be one of molecular mechanisms of aging process.

Older adults demonstrate numerous changes in circadian rhythms compared to younger adults. Indeed, in older subjects, the activity/rest cycle shows a shift towards “morningness”, meaning that older subjects rise from and retire to bed earlier than at a younger age [34]. In turn, this phenomenon affects diurnal variation of recognition memory, reaction time, and other cognitive functions in older humans [35]. In aged mice, activity records showed a clear loss of rhythmicity and circadian amplitude compared to younger animals [36]. Sleep quantity and quality also change with age. Older adults show more wakenings, have longer latencies to fall asleep, and several sleep stages are shorter compared to those of young adults [37,38]. Sleep timing, which is regulated by the interaction of the circadian clock and homeostatic system, is altered in older adults, resulting in a decreased sleep duration at night and an increased daytime sleepiness [39]. Rhythms of core body temperature as well as melatonin and cortisol, the release of which is under the control of SCN, also demonstrate a phase advance and decreased amplitude in the later life [37,40,41], although not all evidence supports the change of their amplitude in healthy aging.

Many metabolic rhythms also exhibit dampening with age. Particularly, there is some evidence that circadian rhythms of blood glucose and lipids change with age [42,43]. Similarly, rhythms of the immune cell number and cytokine secretion are also affected by aging [44]. This might contribute to the development of progressive immunosenescence, a chronic low-grade inflammation, which is in turn associated with metabolic and neurodegenerative disorders [45].

Changes of metabolic rhythms with age are underscored by alterations of clock gene expression rhythms. Chen et al. reported a flattened rhythm and phase advance of the genes *PER1* and *PER2* in human cortex in subjects over 60 years of age [46]. Data on clock gene expression in SCN are inconsistent: Whereas some studies show a smaller amplitude or shorter period of *Period* and *Clock* genes in SCN [47,48], other groups found no difference between young and old rodents [49]. In particular, an elegant work by Nakamura [50] revealed marked reduction of SCN neuronal activity in middle-aged mice relative to young mice, whilst the expression of PER2, a core clock protein, was similar in both age groups, suggesting that the molecular clockwork was not disrupted in the aging mice. A dampening of rhythmic clock gene expression was observed in peripheral tissues in rodents and *Drosophila* [49,51], which suggests an increased susceptibility to aging-related metabolic diseases. Notably, the study of adipose tissue found no blunting of the clock amplitude with aging but still revealed significant differences in the clock gene expression in young and old mice [52].

**Figure 2:**
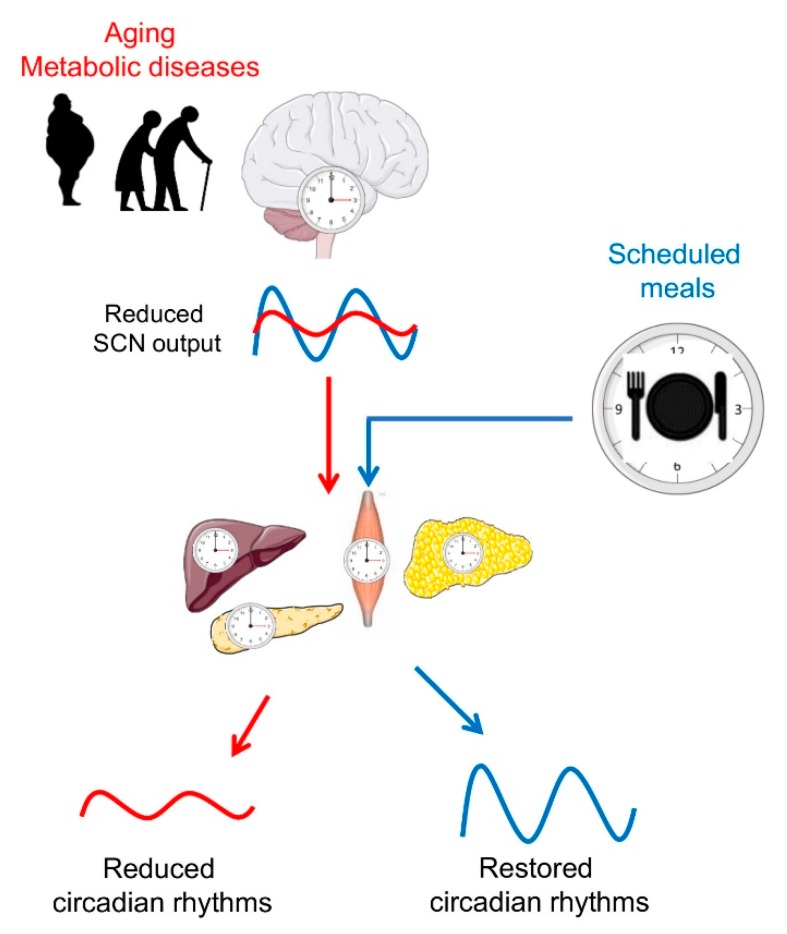
Scheduled meals as an extra-SCN *zeitgeber* for the entrainment of circadian rhythms in aging and metabolic diseases. In later life, a reduced sensitivity of the master clock to light, imbalances of neurotransmitters, and desynchronization of SCN neurons lead to a decrease in the overall amplitude of its firing rhythm. In turn, a weaker SCN output signal reduces the strength of downstream oscillators in central and peripheral tissues. In metabolic diseases such as obesity and type 2 diabetes (T2D), circadian rhythms are also reduced or dysregulated. Providing other *zeitgebers*, such as scheduled meals, which act on the circadian system via extra-SCN pathways, may entrain the circadian system and restore circadian rhythms. Illustrations, used in this Figure, were adapted from Servier Medical Art (http://smart.servier.com/). Servier Medical Art by Servier is licensed under a Creative Commons Attribution 3.0 Unported License.

Aging also alters the capacity of the circadian clock to adjust to jet lag or shift work [53], which could be explained by a reduced responsiveness of the aged master clock to light as well as by a reduced transmission of light through the older eyes [8]. Moreover, aged SCNs show a decreased action of the neurotransmitters arginine vasopressin (AVP), vasoactive intestinal peptide (VIP), and gamma-aminobutyric acid (GABA) [54,55], which results in a loss of neuronal coupling and desynchronization of firing of single neurons. This leads to a dampening of the SCN oscillations and an impaired coordination of peripheral oscillators. In addition, there are some indications for a weakened control by the master clock over the peripheral clock, which obviously contributes to the impaired adaptation to a phase shift of the light/dark schedule in the later life [56] (Figure 2). 

Notably, the responsiveness to nonphotic *zeitgeber* such as temperature rhythm or meal timing is only modestly affected by age. Indeed, environmental temperature rhythms coupled with a light–dark schedule aligned peripheral oscillators with the master clock and improved behavioral rhythms and consolidated sleep in aged *Drosophila* [51]. Older dysrhythmic rats are still able to develop a more consolidated rhythm of anticipatory activity on a restricted feeding schedule [57]. Notably, even mice with a forebrain/SCN-specific *Bmal1* knockout (i.e., mice without a functional master clock) are sufficiently able to selectively resynchronize activity rhythms and oscillations of the peripheral clock gene expression in liver and kidney, but not in other tissues (heart, lung, and spleen), on restricted feeding schedules, suggesting feeding cues differentially entrain peripheral clocks [58]. Notably, locomotor activity and other rhythms might be entrained by mechanisms different from SCN. A range of papers showed that food-anticipatory activity is controlled by a food-entrainable oscillator (FEO). FEO is suggested to comprise a distributed system of clocks that work in concert in response to gastrointestinal input by food including parabrachial nucleus (PBN), nucleus of tractus solitarius (NTS), area postrema (AP), and dorsal nucleus of the vagus (DMX), as well as orexin containing lateral hypothalamus and dorso medial hypothalamus [59,60,61]. Some data showed that aging leads to the decline in mealtime-associated anticipatory behavior, probably resulting from dysfunction in a food-entrainable oscillator [62].

Taken together, animal data suggest that, in older individuals, timed feeding schedules could serve as an effective means of resynchronizing circadian rhythms and mitigating risks of metabolic pathologies involving the circadian system.

## 4. Timing of Eating as an Important Factor of Metabolic Regulation

Because of the tight interaction between the circadian clock and metabolism, meal timing is an important factor of metabolic regulation. Mice, as a nocturnal species, typically consume ~70–80% of their daily food intake during the dark phase. If food availability is restricted to the light phase (i.e., the “wrong” time of the day), circadian oscillators in peripheral tissues uncouple from the central pacemaker in the SCN [63]. This leads to a desynchronization of metabolic processes and increases the risk to develop metabolic diseases. Mice fed an HFD during the light phase gained more weight relative to littermates fed during the dark phase in as little as one week [64]. A reduction in the daily amount of wheel-running has been suggested to contribute to the weight gain [64]. Other reports confirmed that short-term (2 weeks) HFD-feeding at the light phase induces body weight gain but failed to detect significant differences in daily food intake or activity levels [65]. Mice kept under constant bright light or a bright-dim light cycle showed a decreased amplitude of activity rhythms and an increased body weight [66]. Accelerated weight gain, obesity, and changes in metabolic hormones were also seen when mice were housed in 20-h dark/light cycles [67].

Epidemiological studies in humans showed very similar outcomes: Individuals who consumed their meals at the “wrong”/unusual time of the day, as a result of shift work or chronic jet lag, showed an increased risk to develop obesity, type 2 diabetes, and cardiovascular diseases [2,10] (although other cofounding factors such as unhealthy diet, deficit of physical activity, and insufficient sleep may contribute to the risk of adverse metabolic outcomes). Experimental studies confirmed that the misalignment between behavioral cycles (fasting/feeding and sleep/wake cycles) and endogenous circadian cycles induces weight gain and metabolic disruptions in rodents [68] as well as glucose intolerance, deterioration of fatty acid metabolism, and even dysregulation of the circadian transcriptome in humans [69,70,71].

Moreover, in humans, timing of the main meal in the course of the day influences the risk of obesity and success of weight loss therapy. Late lunch eaters lost less weight on a hypocaloric diet than early eaters [72]. Subjects assigned to a high caloric intake during breakfast showed greater weight loss and lower daily glucose, insulin, and ghrelin concentrations and hunger scores than subjects assigned to the same high caloric intake during dinner [73]. Although some epidemiological studies did not find an effect of evening eating on BMI and metabolic parameters [74,75], in most studies, late and delayed eating was associated with weight gain, dysfunction in energy expenditure, and abnormalities in the circadian rhythms of appetite, stress, and sleep hormones [12]. The most extreme case of late/wrong-time eating is the night-eating syndrome, which is also associated with obesity [76]. Furthermore, several experimental studies in humans manipulating the timing of eating among participants [77,78,79,80,81] suggested that delayed eating led to metabolic dysfunction, whereas daytime eating generally improved these parameters. Notably, some findings [82] suggest that an individual’s chronotype is an important factor when prescribing optimal eating times, but this topic is currently poorly investigated.

Moreover, novel studies have suggested that certain time windows are more suitable for the consumption of carbohydrate-rich or fat-rich food to maintain metabolic health. Indeed, mice fed a high fat diet during the end of the active phase had increased body weight and decreased glucose tolerance, compared to mice fed the same diet at the beginning of the active phase [83]. In our recent human study, we investigated whether the consumption of carbohydrates and fat at different times of the day induces different metabolic effects. In a cross-over trial, 29 non-obese men consumed two 4-week isocaloric diets: (1) High-carb meals in the morning and high-fat meals in the afternoon versus (2) the inverse order. This study showed that consumption of high carb meals in the evening induced an unfavorable effect on blood glucose level and glycemic control in subjects with an impaired glucose metabolism [84]. In agreement with this finding, epidemiological studies in humans proposed a beneficial effect of a carbohydrate-rich diet at the beginning of the day, which is shown to be protective against the development of diabetes and metabolic syndrome [85,86]. Moreover, we found that timing of carbohydrates and fat intake also affected average daily blood concentrations of the adipokines leptin and visfatin [87].

A range of studies provides potential explanations for why timing of meal intake induces different effects depending on the time of day. As a result of circadian regulation, humans show a better insulin sensitivity, beta cell responsiveness and glucose tolerance, and an increased postprandial thermogenesis in the morning than in the afternoon/evening [69,84,88]. We and others showed that the consumption of the same meal in the morning and in the evening induces different postprandial glucose concentrations and altered secretions of insulin, C-peptide, and of the incretins glucagon-like peptide 1 (GLP1) and gastric inhibitory polypeptide (GIP) [84,89,90]. These data suggest that early eating is in alignment with metabolic rhythms and therefore beneficial for metabolic health.

Notably, insulin and oxyntomodulin (and possibly other meal-induced hormones and humoral stimuli) are involved in the circadian entrainment of liver and adipose tissue [91,92], which may explain the effects feeding imposes on circadian rhythms. Moreover, the postprandial increase of various nutrients such as glucose, lipids, and amino acids affects the circadian clock via key intracellular metabolic sensors such as SIRT1, mTOR, S6K, AMPK, PPARs, RORs, and Rev-Erbs [28], and a combination of nutrients might influence the clock in different ways than individual nutrients. The recent elegant work of Mukherij et al. confirmed a central role of PPARα, Rev-Erbα, and CREB in dietary-induced changes of peripheral circadian rhythms [93]. 

## 5. Time-Restricted Feeding as a Promising Tool for Circadian and Metabolic Improvements

While it is commonly assumed that most people eat breakfast, lunch, and dinner, and a couple of snacks, the reality is quite different. Using a smartphone app, Gill and Panda [94] revealed a frequent and erratic daily eating pattern of healthy American adults, with up to 11 eating events per day, 25% of intake occurring before noon, and 35% of intake occurring after 18:00 h. More than half of the adults eat over a period of 15 h or longer every day, and this eating period is often shifted to a later time on weekends, indicating a “social jet lag” [95]. A study in Indian adults confirmed a high number of eating events and long eating period of at least 15 h per day, with more than a third of intake occurring after 18:00 h [96]. Interestingly, overweight individuals with initially >14 h eating duration who reduced their eating duration to a self-selected window of 10–12 h showed sustained weight loss, felt more energetic, and reported more sleep satisfaction after 16 weeks and one year of the intervention [94]. These data suggest that a shortening of the eating period might have beneficial effects on metabolic parameters in humans. One possible explanation of this effect might be the elongation of the fasting period (typically beyond 12 h) which leads to the depletion of liver glycogen stores and a metabolic switch from lipid/cholesterol synthesis and fat storage to mobilization of fat through fatty acid oxidation and fatty acid-derived ketones [97].

First data on metabolic effects of “time-restricted feeding” (TRF) were collected in rodent studies. “Time-restricted feeding” is a term often used in chronobiology, which means the restriction of the food access to anywhere between 2 and 12 h during the day or night [98]. If the period of food access is <6 h, animals cannot eat the same amount of food as ad libitum animals; however, if the period of food access is >8 h, the amount of food consumed in the TRF group almost equals the amount in the ad libitum group; therefore, this length is often used in experiments with TRF. While restricting feeding to the “wrong” circadian phase induces adverse metabolic effects as described above, restricting feeding to the “right” circadian phase (i.e., dark phase in rodents) is shown to be protective against metabolic disturbances induced by obesogenic diets. Compared with ad libitum feeding, TRF during the dark phase increases the amplitude of circadian rhythms and is protective against HFD-induced obesity, glucose intolerance, leptin resistance, hepatic steatosis, and tissue inflammation [22,24]. Interestingly, metabolic benefits were proportional to the fasting duration [22]. Furthermore, the protective effect was maintained even when TRF was temporarily interrupted by ad libitum access to food on two days during the week imitating weekend habits relevant to human lifestyle [22]. The beneficial effect of the dark phase TRF was not only shown for HFD but also for high-sucrose and high-fructose diet, and even in mice with pre-existing obesity and metabolic disturbances [22]. Interestingly, genetic models of obesity in rats showing blunted circadian activity rhythm reduced weight gain upon the dark phase TRF [99].

In humans, the “16:8 diet” with an eating period of 8h a day (as well as other forms of intermittent fasting described below) is an increasingly popular dietary approach used for weight loss and overall health [100]. However, thoughtful investigation of its metabolic effects is needed. So far, six clinical trials on TRF in humans with eating periods 4–10 h have been published. In line with the abovementioned data, effects of TRF in humans depend on the day time of the eating period. In the study of Sutton et al. [101], men with prediabetes were randomized to early TRF (6-h eating period with dinner before 3 p.m.) or a control schedule (12-h eating period) for five weeks and were strongly controlled to prevent weight loss. Nevertheless, participants slightly lost weight in both study arms (−1.4 kg *vs*. −1.0 kg) without difference between the two groups. This study showed that restricting food intake to the morning resulted in an improvement of insulin sensitivity, beta-cell responsiveness, blood pressure, inflammation, oxidative stress, and appetite [101].

LeCheminant et al. [78] studied young men who were either prohibited from eating between 1900 h–0600 h or ate as per their usual schedule for two weeks. Refraining from eating after 1900 h led to a small reduction in caloric intake and weight (−0.4 kg *vs*. +0.6 kg upon usual schedule) but did not clarify the role of meal timing in the found effect. In the study of Moro et al. [102], healthy resistance-trained men were assigned to isocaloric TRF (three meals consumed within a 8-h period at 1 p.m., 4 p.m. and 8 p.m.) or normal diet group (three meals consumed within a 16-h period at 8 a.m., 1 p.m. and 8 p.m.) for eight weeks. The TRF diet led to the reduction of body fat (but maintenance of muscle mass), increase of adiponectin, and decrease of leptin and triglycerides, whereas no difference between diets was detected for glucose, insulin, total cholesterol, high-density lipoprotein, and low-density lipoprotein. Gabel et al. [103] also compared an 8-h TRF (ad libitum eating between 10:00 to 18:00 h) for 12 weeks in obese subjects with a control group which was instructed to maintain their weight without changing their eating habits. Body weight (–2.6%), energy intake, and systolic blood pressure decreased in the TRF group, but other anthropometric, glucose, and lipid parameters did not differ between the groups. Thus, three studies in which food intake was restricted to the middle of the day but was not precisely matched resulted in a reduced body weight or fat mass, with contradictory results concerning fasting glucose, insulin, and lipids [94,102,103]. 

Studies of Carlson et al. [104] and Stote et al. [105] compared the metabolic effects of 3 meals/day with 1 large meal/day which was consumed in the early evening between 5 p.m. and 9.00 p.m. and contained the same amount of calories. Healthy subjects consuming 1 meal/day for eight weeks showed a reduction in fat mass and increases in blood pressure and in total, high-density lipoprotein, and low-density lipoprotein cholesterol [105]. In addition, they exhibited elevated fasting glucose levels, impaired morning glucose tolerance associated with a delayed insulin response, and increased postprandial ghrelin levels [104]. Fasting levels of insulin, leptin, ghrelin, and adiponectin were not significantly affected by meal frequency [104]. Tinsley et al. [106] investigated the effects of a similar diet as in the abovementioned studies in young men performing resistance training. Four days per week, participants were required to consume all calories in any four-hour window between 4 p.m. and midnight. TRF reduced energy intake by ∼650 kcal per day but did not affect total body composition within eight weeks of intervention. Thus, restricting food intake to the late afternoon or evening (after 4 p.m.) did not change metabolic state or even worsened postprandial glucose, beta-cell responsiveness, blood pressure, and lipid levels [104,105,106]. These data suggest that, in humans, restricting the eating window to the early time of the day will expectedly induce beneficial metabolic effects in contrast to a delayed eating window. However, most of the published human TRF studies did not carefully monitor calorie intake (which led to weight loss, and this makes it difficult to interpret the TRF and weight loss effects separately), macronutrient content, activity levels, and timing of sleep-wake cycles and used small sample sizes. 

Taken together, TRF (especially early TRF) represents a promising dietary approach for the prevention and therapy of metabolic disturbances. It deemphasizes caloric intake, making it an attractive and easily adoptable lifestyle modification. Providing other *zeitgebers* such as scheduled meals, which act on the circadian system via extra-SCN pathways, may entrain the circadian system and restore circadian rhythms. In older subjects, TRF might be used to restore disturbed circadian rhythms and improve metabolic health (Figure 2). However, to detect an impact of dietary regimen, precisely assessment of circadian rhythms has to be performed in study subjects before scheduling the meal time, and the development of corresponding tools is needed. Recently, a group of Achim Kramer developed an assay (BodyTime) to estimate the internal circadian time in humans from a single blood sample based on the expression levels of a gene set [107]. Whether this assay is accurate and sensitive enough to detect changes of circadian clock upon TRF intervention has to be investigated in future studies. Moreover, further experimental studies in humans are needed to investigate effects of TRF: (i) Without weight loss; (ii) in long-term studies; (iii) in diverse populations (i.e., subjects with overweight, obesity, diabetes, in subjects of different age); and (iv) including analyses of molecular mechanisms underlying the TRF-induced changes.

## 6. Other Dietary Approaches Affecting the Circadian Clock

TRF is only one form of intermittent fasting (IF), which means voluntary abstinence from food for different periods of time [108]. IF includes complete alternate day fasting (involving a combination of no-eating days with eating days), modified fasting (consumption of 20–25% of energy needs on scheduled fasting days), and different fasting regimes for religious purposes. Most of the IF data are based on animal models. IF-fed animals showed improved glucose metabolism, resistance to cardiovascular diseases and cancer, and increased life span [109,110]. IF effects on circadian rhythms depended on the day time of the food intake. If food was provided at the inactive phase, mice showed arrhythmic clock gene expression in the liver, whereas feeding at the active phase resulted in rhythms similar to ad libitum feeding [111]. In humans, most studies showed reductions in glucose and insulin concentrations, improvement of lipid levels, and reductions in inflammatory factors, although different fasting regimens made the data very heterogeneous [100]. Beneficial effects of IF could be particularly explained by weight loss (found in most IF studies) and a prolonged fasting time; however, exact mechanisms affecting circadian rhythms need further investigation.

Further, calorie restriction (CR) is another dietary regimen affecting circadian rhythms and aging processes. CR means limiting the daily caloric intake to 60–70% of ad libitum intake. In animal studies, CR prevents or delays the development of age-related pathologies such as kidney disease, cancer, and diabetes and extends the life span [112,113]. In humans, long-term CR improves the level of risk factors contributing to the development of T2D, inflammation, and atherosclerosis [114]. In addition to body weight reduction, other mechanisms are considered to contribute to this phenomenon [112,113]. In particular, CR reduces oxidative stress and induces appropriate autophagy, which increases the life span [115]. A recent study in Drosophila showed that lifespan-extending effects of CR are mediated by an increased amplitude of clock genes regulating lipid metabolism [116]. Several mouse studies confirmed that CR synchronizes the peripheral clock and can also affect the SCN clock [117,118], which leads to synchronization of biochemical processes and metabolic functions and results in life span extension. Notably, CR effects on clock rhythms could be particularly explained by mechanisms which are involved in TRF action. Indeed, CR-fed animals usually consume most of their daily food amount within a short period of time, which leads to clock synchronization [31]. CR-mediated effects on clock in mice are mediated by BMAL1-dependent and -independent mechanisms [119]. Similar to rodents, a hypocaloric diet induces alterations of the clock gene expression in human adipose tissue [120], but mechanisms of this effect have not been investigated in humans. 

## 7. Conclusions

Dietary approaches based on meal timing are a promising strategy for the modulation of circadian rhythms and clock-controlled metabolic functions in humans. Especially TRF represents an attractive and easy-to-adapt tool for the prevention and therapy of metabolic disturbances. In older subjects, timed dietary approaches might be used to restore disturbed circadian rhythms and to improve metabolic health. However, in view of the complexity of clock–metabolism interactions, future carefully controlled studies are needed to elucidate dietary effects on circadian rhythms in humans and underlying molecular mechanisms.

## Abbreviations

BMI	Body mass index
CR	Calorie restriction
FEO	Food-entrainable oscillator
HFD	High-fat diet
IF	Intermittent fasting
T2D	Type 2 diabetes
TRF	Time-restricted feeding

## References

[1] Panda S. (2016). Circadian physiology of metabolism. Science.

[2] Antunes L.C., Levandovski R., Dantas G., Caumo W., Hidalgo M.P. (2010). Obesity and shift work: Chronobiological aspects. Nutr. Res. Rev..

[3] De Bacquer D., Van Risseghem M., Clays E., Kittel F., De Backer G., Braeckman L. (2009). Rotating shift work and the metabolic syndrome: A prospective study. Int. J. Epidemiol..

[4] Vetter C., Devore E.E., Wegrzyn L.R., Massa J., Speizer F.E., Kawachi I., Rosner B., Stampfer M.J., Schernhammer E.S. (2016). Association Between Rotating Night Shift Work and Risk of Coronary Heart Disease Among Women. JAMA J. Am. Med. Assoc..

[5] Ando H., Takamura T., Matsuzawa-Nagata N., Shima K.R., Eto T., Misu H., Shiramoto M., Tsuru T., Irie S., Fujimura A. (2009). Clock gene expression in peripheral leucocytes of patients with type 2 diabetes. Diabetologia.

[6] Vieira E., Ruano E., Figueroa A.L., Aranda G., Momblan D., Carmona F., Gomis R., Vidal J., Hanzu F.A. (2014). Altered clock gene expression in obese visceral adipose tissue is associated with metabolic syndrome. PloS ONE.

[7] Gomez-Abellan P., Hernandez-Morante J.J., Lujan J.A., Madrid J.A., Garaulet M. (2008). Clock genes are implicated in the human metabolic syndrome. Int. J. Obes..

[8] Hood S., Amir S. (2017). The aging clock: Circadian rhythms and later life. J. Clin. Investig..

[9] Niccoli T., Partridge L. (2012). Ageing as a risk factor for disease. Curr. Biol..

[10] Jiang P., Turek F.W. (2017). Timing of meals: When is as critical as what and how much. Am. J. Physiol. Endocrinol. Metab..

[11] Asher G., Sassone-Corsi P. (2015). Time for food: The intimate interplay between nutrition, metabolism, and the circadian clock. Cell.

[12] Allison K.C., Goel N. (2018). Timing of eating in adults across the weight spectrum: Metabolic factors and potential circadian mechanisms. Physiol. Behav..

[13] Johnston J.D., Ordovas J.M., Scheer F.A., Turek F.W. (2016). Circadian Rhythms, Metabolism, and Chrononutrition in Rodents and Humans. Adv. Nutr..

[14] Garrido M., Terron M.P., Rodriguez A.B. (2013). Chrononutrition against oxidative stress in aging. Oxidative Med. Cell. Longev..

[15] Brown S.A. (2016). Circadian Metabolism: From Mechanisms to Metabolomics and Medicine. Trends Endocrinol. Metab..

[16] Keller M., Mazuch J., Abraham U., Eom G.D., Herzog E.D., Volk H.D., Kramer A., Maier B. (2009). A circadian clock in macrophages controls inflammatory immune responses. Proc. Natl. Acad. Sci. USA.

[17] Eckel-Mahan K.L., Patel V.R., de Mateo S., Orozco-Solis R., Ceglia N.J., Sahar S., Dilag-Penilla S.A., Dyar K.A., Baldi P., Sassone-Corsi P. (2013). Reprogramming of the circadian clock by nutritional challenge. Cell.

[18] Wang J., Mauvoisin D., Martin E., Atger F., Galindo A.N., Dayon L., Sizzano F., Palini A., Kussmann M., Waridel P. (2017). Nuclear Proteomics Uncovers Diurnal Regulatory Landscapes in Mouse Liver. Cell Metab..

[19] Turek F.W. (2005). Obesity and metabolic syndrome in circadian Clock mutant mice. Science.

[20] Paschos G.K., Ibrahim S., Song W.L., Kunieda T., Grant G., Reyes T.M., Bradfield C.A., Vaughan C.H., Eiden M., Masoodi M. (2012). Obesity in mice with adipocyte-specific deletion of clock component Arntl. Nat. Med..

[21] Lamia K.A., Storch K.F., Weitz C.J. (2008). Physiological significance of a peripheral tissue circadian clock. Proc. Natl. Acad. Sci. USA.

[22] Chaix A., Zarrinpar A., Miu P., Panda S. (2014). Time-restricted feeding is a preventative and therapeutic intervention against diverse nutritional challenges. Cell Metab..

[23] Kohsaka A., Laposky A.D., Ramsey K.M., Estrada C., Joshu C., Kobayashi Y., Turek F.W., Bass J. (2007). High-fat diet disrupts behavioral and molecular circadian rhythms in mice. Cell Metab..

[24] Hatori M., Vollmers C., Zarrinpar A., DiTacchio L., Bushong E.A., Gill S., Leblanc M., Chaix A., Joens M., Fitzpatrick J.A. (2012). Time-restricted feeding without reducing caloric intake prevents metabolic diseases in mice fed a high-fat diet. Cell Metab..

[25] Su W., Xie Z., Guo Z., Duncan M.J., Lutshumba J., Gong M.C. (2012). Altered clock gene expression and vascular smooth muscle diurnal contractile variations in type 2 diabetic db/db mice. Am. J. Physiol..

[26] Ando H., Kumazaki M., Motosugi Y., Ushijima K., Maekawa T., Ishikawa E., Fujimura A. (2011). Impairment of peripheral circadian clocks precedes metabolic abnormalities in ob/ob mice. Endocrinology.

[27] Pivovarova O., Jurchott K., Rudovich N., Hornemann S., Ye L., Mockel S., Murahovschi V., Kessler K., Seltmann A.C., Maser-Gluth C. (2015). Changes of Dietary Fat and Carbohydrate Content Alter Central and Peripheral Clock in Humans. J. Clin. Endocrinol. Metab..

[28] Chaudhari A., Gupta R., Makwana K., Kondratov R. (2017). Circadian clocks, diets and aging. Nutr. Healthy Aging.

[29] Davidson A.J. (2006). Chronic jet-lag increases mortality in aged mice. Curr. Biol..

[30] Yu E.A., Weaver D.R. (2011). Disrupting the circadian clock: Gene-specific effects on aging, cancer, and other phenotypes. Aging.

[31] Froy O. (2011). Circadian rhythms, aging, and life span in mammals. Physiology.

[32] Froy O., Chapnik N., Miskin R. (2006). Long-lived alphaMUPA transgenic mice exhibit pronounced circadian rhythms. Am. J. Physiol..

[33] Fonseca Costa S.S., Ripperger J.A. (2015). Impact of the circadian clock on the aging process. Front. Neurol..

[34] Roenneberg T., Kuehnle T., Juda M., Kantermann T., Allebrandt K., Gordijn M., Merrow M. (2007). Epidemiology of the human circadian clock. Sleep Med. Rev..

[35] Schmidt C., Peigneux P., Cajochen C., Collette F. (2012). Adapting test timing to the sleep-wake schedule: Effects on diurnal neurobehavioral performance changes in young evening and older morning chronotypes. Chronobiol. Int..

[36] Banks G., Nolan P.M., Peirson S.N. (2016). Reciprocal interactions between circadian clocks and aging. Mamm. Genome.

[37] Dijk D.J., Duffy J.F., Czeisler C.A. (2000). Contribution of circadian physiology and sleep homeostasis to age-related changes in human sleep. Chronobiol. Int..

[38] Hayashi Y., Endo S. (1982). All-night sleep polygraphic recordings of healthy aged persons: REM and slow-wave sleep. Sleep.

[39] Huang Y.L., Liu R.Y., Wang Q.S., Van Someren E.J., Xu H., Zhou J.N. (2002). Age-associated difference in circadian sleep-wake and rest-activity rhythms. Physiol. Behav..

[40] Touitou Y., Fevre M., Lagoguey M., Carayon A., Bogdan A., Reinberg A., Beck H., Cesselin F., Touitou C. (1981). Age- and mental health-related circadian rhythms of plasma levels of melatonin, prolactin, luteinizing hormone and follicle-stimulating hormone in man. J. Endocrinol..

[41] Van Cauter E., Leproult R., Kupfer D.J. (1996). Effects of gender and age on the levels and circadian rhythmicity of plasma cortisol. J. Clin. Endocrinol. Metab..

[42] Wijsman C.A., van Heemst D., Hoogeveen E.S., Slagboom P.E., Maier A.B., de Craen A.J., van der Ouderaa F., Pijl H., Westendorp R.G., Mooijaart S.P. (2013). Ambulant 24-h glucose rhythms mark calendar and biological age in apparently healthy individuals. Aging Cell.

[43] Singh R., Singh R.K., Masood T., Tripathi A.K., Mahdi A.A., Singh R.K., Schwartzkopff O., Cornelissen G. (2015). Circadian time structure of circulating plasma lipid peroxides, antioxidant enzymes and other small molecules in peptic ulcers. Clin. Chim. Acta.

[44] Mazzoccoli G., Inglese M., De Cata A., Carughi S., Dagostino M.P., Marzulli N., Damato M., Grilli M., Giuliani F., Greco A. (2011). Neuroendocrine-immune interactions in healthy aging. Geriatr. Gerontol. Int..

[45] Deleidi M., Jaggle M., Rubino G. (2015). Immune aging, dysmetabolism, and inflammation in neurological diseases. Front. Neurosci..

[46] Chen C.Y., Logan R.W., Ma T., Lewis D.A., Tseng G.C., Sibille E., McClung C.A. (2016). Effects of aging on circadian patterns of gene expression in the human prefrontal cortex. Proc. Natl. Acad. Sci. USA.

[47] Bonaconsa M., Malpeli G., Montaruli A., Carandente F., Grassi-Zucconi G., Bentivoglio M. (2014). Differential modulation of clock gene expression in the suprachiasmatic nucleus, liver and heart of aged mice. Exp. Gerontol..

[48] Kolker D.E., Fukuyama H., Huang D.S., Takahashi J.S., Horton T.H., Turek F.W. (2003). Aging alters circadian and light-induced expression of clock genes in golden hamsters. J. Biol. Rhythm..

[49] Yamazaki S., Straume M., Tei H., Sakaki Y., Menaker M., Block G.D. (2002). Effects of aging on central and peripheral mammalian clocks. Proc. Natl. Acad. Sci. USA.

[50] Nakamura T.J., Nakamura W., Yamazaki S., Kudo T., Cutler T., Colwell C.S., Block G.D. (2011). Age-related decline in circadian output. J. Neurosci..

[51] Luo W., Chen W.F., Yue Z., Chen D., Sowcik M., Sehgal A., Zheng X. (2012). Old flies have a robust central oscillator but weaker behavioral rhythms that can be improved by genetic and environmental manipulations. Aging Cell.

[52] Sutton G.M., Ptitsyn A.A., Floyd Z.E., Yu G., Wu X., Hamel K., Shah F.S., Centanni A., Eilertsen K., Kheterpal I. (2013). Biological aging alters circadian mechanisms in murine adipose tissue depots. Age.

[53] Monk T.H., Buysse D.J., Carrier J., Kupfer D.J. (2000). Inducing jet-lag in older people: Directional asymmetry. J. Sleep Res..

[54] Hofman M.A., Swaab D.F. (1994). Alterations in circadian rhythmicity of the vasopressin-producing neurons of the human suprachiasmatic nucleus (SCN) with aging. Brain Res..

[55] Palomba M., Nygard M., Florenzano F., Bertini G., Kristensson K., Bentivoglio M. (2008). Decline of the presynaptic network, including GABAergic terminals, in the aging suprachiasmatic nucleus of the mouse. J. Biol. Rhythm..

[56] Sellix M.T., Evans J.A., Leise T.L., Castanon-Cervantes O., Hill D.D., DeLisser P., Block G.D., Menaker M., Davidson A.J. (2012). Aging differentially affects the re-entrainment response of central and peripheral circadian oscillators. J. Neurosci..

[57] Walcott E.C., Tate B.A. (1996). Entrainment of aged, dysrhythmic rats to a restricted feeding schedule. Physiol. Behav..

[58] Izumo M., Pejchal M., Schook A.C., Lange R.P., Walisser J.A., Sato T.R., Wang X., Bradfield C.A., Takahashi J.S. (2014). Differential effects of light and feeding on circadian organization of peripheral clocks in a forebrain Bmal1 mutant. Elife.

[59] Juarez C., Morgado E., Waliszewski S.M., Martinez A.J., Meza E., Caba M. (2012). Synchronization of PER1 protein in parabrachial nucleus in a natural model of food anticipatory activity. Eur. J. Neurosci..

[60] Yamanaka A., Tsunematsu T. (2010). New approaches for the study of orexin function. J. Neuroendocrinol..

[61] Mieda M., Williams S.C., Richardson J.A., Tanaka K., Yanagisawa M. (2006). The dorsomedial hypothalamic nucleus as a putative food-entrainable circadian pacemaker. Proc. Natl. Acad. Sci. USA.

[62] Tanaka Y., Kurasawa M., Nakamura K. (2000). Recovery of diminished mealtime-associated anticipatory behavior by aniracetam in aged rats. Pharmacol. Biochem. Behav..

[63] Damiola F. (2000). Restricted feeding uncouples circadian oscillators in peripheral tissues from the central pacemaker in the suprachiasmatic nucleus. Genes Dev..

[64] Yasumoto Y., Hashimoto C., Nakao R., Yamazaki H., Hiroyama H., Nemoto T., Yamamoto S., Sakurai M., Oike H., Wada N. (2016). Short-term feeding at the wrong time is sufficient to desynchronize peripheral clocks and induce obesity with hyperphagia, physical inactivity and metabolic disorders in mice. Metab. Clin. Exp..

[65] Arble D.M., Bass J., Laposky A.D., Vitaterna M.H., Turek F.W. (2009). Circadian timing of food intake contributes to weight gain. Obesity.

[66] Fonken L.K., Workman J.L., Walton J.C., Weil Z.M., Morris J.S., Haim A., Nelson R.J. (2010). Light at night increases body mass by shifting the time of food intake. Proc. Natl. Acad. Sci. USA.

[67] Karatsoreos I.N., Bhagat S., Bloss E.B., Morrison J.H., McEwen B.S. (2011). Disruption of circadian clocks has ramifications for metabolism, brain, and behavior. Proc. Natl. Acad. Sci. USA.

[68] Salgado-Delgado R., Angeles-Castellanos M., Saderi N., Buijs R.M., Escobar C. (2010). Food intake during the normal activity phase prevents obesity and circadian desynchrony in a rat model of night work. Endocrinology.

[69] Scheer F.A., Hilton M.F., Mantzoros C.S., Shea S.A. (2009). Adverse metabolic and cardiovascular consequences of circadian misalignment. Proc. Natl. Acad. Sci. USA.

[70] Archer S.N., Laing E.E., Moller-Levet C.S., van der Veen D.R., Bucca G., Lazar A.S., Santhi N., Slak A., Kabiljo R., von Schantz M. (2014). Mistimed sleep disrupts circadian regulation of the human transcriptome. Proc. Natl. Acad. Sci. USA.

[71] Wefers J., van Moorsel D., Hansen J., Connell N.J., Havekes B., Hoeks J., van Marken Lichtenbelt W.D., Duez H., Phielix E., Kalsbeek A. (2018). Circadian misalignment induces fatty acid metabolism gene profiles and compromises insulin sensitivity in human skeletal muscle. Proc. Natl. Acad. Sci. USA.

[72] Garaulet M., Gomez-Abellan P., Alburquerque-Bejar J.J., Lee Y.C., Ordovas J.M., Scheer F.A. (2013). Timing of food intake predicts weight loss effectiveness. Int. J. Obes..

[73] Jakubowicz D., Barnea M., Wainstein J., Froy O. (2013). High caloric intake at breakfast vs. dinner differentially influences weight loss of overweight and obese women. Obesity.

[74] Sandhu S.K., Tang T.S. (2017). When′s dinner? Does timing of dinner affect the cardiometabolic risk profiles of South-Asian Canadians at risk for diabetes. Diabet. Med..

[75] Aljuraiban G.S., Chan Q., Oude Griep L.M., Brown I.J., Daviglus M.L., Stamler J., Van Horn L., Elliott P., Frost G.S., Group I.R. (2015). The impact of eating frequency and time of intake on nutrient quality and Body Mass Index: The INTERMAP Study, a Population-Based Study. J. Acad. Nutr. Diet..

[76] Gallant A.R., Lundgren J., Drapeau V. (2012). The night-eating syndrome and obesity. Obes. Rev..

[77] Wehrens S.M.T., Christou S., Isherwood C., Middleton B., Gibbs M.A., Archer S.N., Skene D.J., Johnston J.D. (2017). Meal Timing Regulates the Human Circadian System. Curr. Biol..

[78] LeCheminant J.D., Christenson E., Bailey B.W., Tucker L.A. (2013). Restricting night-time eating reduces daily energy intake in healthy young men: A short-term cross-over study. Br. J. Nutr..

[79] Hibi M., Masumoto A., Naito Y., Kiuchi K., Yoshimoto Y., Matsumoto M., Katashima M., Oka J., Ikemoto S. (2013). Nighttime snacking reduces whole body fat oxidation and increases LDL cholesterol in healthy young women. Am. J. Physiol..

[80] Bandin C., Scheer F.A., Luque A.J., Avila-Gandia V., Zamora S., Madrid J.A., Gomez-Abellan P., Garaulet M. (2015). Meal timing affects glucose tolerance, substrate oxidation and circadian-related variables: A randomized, crossover trial. Int. J. Obes..

[81] Qin L.Q., Li J., Wang Y., Wang J., Xu J.Y., Kaneko T. (2003). The effects of nocturnal life on endocrine circadian patterns in healthy adults. Life Sci..

[82] McHill A.W., Phillips A.J., Czeisler C.A., Keating L., Yee K., Barger L.K., Garaulet M., Scheer F.A., Klerman E.B. (2017). Later circadian timing of food intake is associated with increased body fat. Am. J. Clin. Nutr..

[83] Bray M.S. (2014). Time-of-day-dependent dietary fat consumption influences multiple cardiometabolic syndrome parameters in mice. Int. J. Obes..

[84] Kessler K., Hornemann S., Petzke K.J., Kemper M., Kramer A., Pfeiffer A.F., Pivovarova O., Rudovich N. (2017). The effect of diurnal distribution of carbohydrates and fat on glycaemic control in humans: A randomized controlled trial. Sci. Rep..

[85] Almoosawi S., Prynne C.J., Hardy R., Stephen A.M. (2013). Diurnal eating rhythms: Association with long-term development of diabetes in the 1946 British birth cohort. Nutr. Metab. Cardiovasc. Dis..

[86] Almoosawi S., Prynne C.J., Hardy R., Stephen A.M. (2013). Time-of-day and nutrient composition of eating occasions: Prospective association with the metabolic syndrome in the 1946 British birth cohort. Int. J. Obes..

[87] Kessler K., Hornemann S., Petzke K.J., Kemper M., Markova M., Rudovich N., Grune T., Kramer A., Pfeiffer A.F.H., Pivovarova-Ramich O. (2018). Diurnal distribution of carbohydrates and fat affects substrate oxidation and adipokine secretion in humans. Am. J. Clin. Nutr.

[88] Bo S., Fadda M., Castiglione A., Ciccone G., De Francesco A., Fedele D., Guggino A., Parasiliti Caprino M., Ferrara S., Vezio Boggio M. (2015). Is the timing of caloric intake associated with variation in diet-induced thermogenesis and in the metabolic pattern? A randomized cross-over study. Int. J. Obes..

[89] Jakubowicz D., Wainstein J., Ahren B., Bar-Dayan Y., Landau Z., Rabinovitz H.R., Froy O. (2015). High-energy breakfast with low-energy dinner decreases overall daily hyperglycaemia in type 2 diabetic patients: A randomised clinical trial. Diabetologia.

[90] Lindgren O., Mari A., Deacon C.F., Carr R.D., Winzell M.S., Vikman J., Ahren B. (2009). Differential islet and incretin hormone responses in morning versus afternoon after standardized meal in healthy men. J. Clin. Endocrinol. Metab..

[91] Sato M., Murakami M., Node K., Matsumura R., Akashi M. (2014). The role of the endocrine system in feeding-induced tissue-specific circadian entrainment. Cell Rep..

[92] Landgraf D., Tsang A.H., Leliavski A., Koch C.E., Barclay J.L., Drucker D.J., Oster H. (2015). Oxyntomodulin regulates resetting of the liver circadian clock by food. Elife.

[93] Mukherji A., Kobiita A., Chambon P. (2015). Shifting the feeding of mice to the rest phase creates metabolic alterations, which, on their own, shift the peripheral circadian clocks by 12 h. Proc. Natl. Acad. Sci. USA.

[94] Gill S., Panda S. (2015). A Smartphone App Reveals Erratic Diurnal Eating Patterns in Humans that Can Be Modulated for Health Benefits. Cell Metab..

[95] Roenneberg T., Allebrandt K.V., Merrow M., Vetter C. (2012). Social jetlag and obesity. Curr. Biol..

[96] Gupta N.J., Kumar V., Panda S. (2017). A camera-phone based study reveals erratic eating pattern and disrupted daily eating-fasting cycle among adults in India. PloS ONE.

[97] Anton S.D., Moehl K., Donahoo W.T., Marosi K., Lee S.A., Mainous A.G., Leeuwenburgh C., Mattson M.P. (2018). Flipping the Metabolic Switch: Understanding and Applying the Health Benefits of Fasting. Obesity.

[98] Manoogian E.N.C., Panda S. (2017). Circadian rhythms, time-restricted feeding, and healthy aging. Ageing Res. Rev..

[99] Mistlberger R.E., Lukman H., Nadeau B.G. (1998). Circadian rhythms in the Zucker obese rat: Assessment and intervention. Appetite.

[100] Patterson R.E., Sears D.D. (2017). Metabolic Effects of Intermittent Fasting. Annu Rev. Nutr..

[101] Sutton E.F., Beyl R., Early K.S., Cefalu W.T., Ravussin E., Peterson C.M. (2018). Early Time-Restricted Feeding Improves Insulin Sensitivity, Blood Pressure, and Oxidative Stress Even without Weight Loss in Men with Prediabetes. Cell Metab..

[102] Moro T., Tinsley G., Bianco A., Marcolin G., Pacelli Q.F., Battaglia G., Palma A., Gentil P., Neri M., Paoli A. (2016). Effects of eight weeks of time-restricted feeding (16/8) on basal metabolism, maximal strength, body composition, inflammation, and cardiovascular risk factors in resistance-trained males. J. Transl. Med..

[103] Gabel K., Hoddy K.K., Haggerty N., Song J., Kroeger C.M., Trepanowski J.F., Panda S., Varady K.A. (2018). Effects of 8-h time restricted feeding on body weight and metabolic disease risk factors in obese adults: A pilot study. Nutr. Healthy Aging.

[104] Carlson O., Martin B., Stote K.S., Golden E., Maudsley S., Najjar S.S., Ferrucci L., Ingram D.K., Longo D.L., Rumpler W.V. (2007). Impact of reduced meal frequency without caloric restriction on glucose regulation in healthy, normal-weight middle-aged men and women. Metab. Clin. Exp..

[105] Stote K.S., Baer D.J., Spears K., Paul D.R., Harris G.K., Rumpler W.V., Strycula P., Najjar S.S., Ferrucci L., Ingram D.K. (2007). A controlled trial of reduced meal frequency without caloric restriction in healthy, normal-weight, middle-aged adults. Am. J. Clin. Nutr..

[106] Tinsley G.M., Forsse J.S., Butler N.K., Paoli A., Bane A.A., La Bounty P.M., Morgan G.B., Grandjean P.W. (2017). Time-restricted feeding in young men performing resistance training: A randomized controlled trial. Eur. J. Sport Sci..

[107] Wittenbrink N., Ananthasubramaniam B., Munch M., Koller B., Maier B., Weschke C., Bes F., de Zeeuw J., Nowozin C., Wahnschaffe A. (2018). High-accuracy determination of internal circadian time from a single blood sample. J. Clin. Investig..

[108] Patterson R.E., Laughlin G.A., LaCroix A.Z., Hartman S.J., Natarajan L., Senger C.M., Martinez M.E., Villasenor A., Sears D.D., Marinac C.R. (2015). Intermittent Fasting and Human Metabolic Health. J. Acad. Nutr. Diet..

[109] Anson R.M., Guo Z., de Cabo R., Iyun T., Rios M., Hagepanos A., Ingram D.K., Lane M.A., Mattson M.P. (2003). Intermittent fasting dissociates beneficial effects of dietary restriction on glucose metabolism and neuronal resistance to injury from calorie intake. Proc. Natl. Acad. Sci. USA.

[110] Mattson M.P., Wan R. (2005). Beneficial effects of intermittent fasting and caloric restriction on the cardiovascular and cerebrovascular systems. J. Nutr. Biochem..

[111] Froy O., Chapnik N., Miskin R. (2009). Effect of intermittent fasting on circadian rhythms in mice depends on feeding time. Mech. Ageing Dev..

[112] Masoro E.J. (2009). Caloric restriction-induced life extension of rats and mice: A critique of proposed mechanisms. Biochim. Biophys. Acta.

[113] Fontana L., Nehme J., Demaria M. (2018). Caloric restriction and cellular senescence. Mech. Ageing Dev..

[114] Fontana L. (2009). Modulating human aging and age-associated diseases. Biochim. Biophys. Acta.

[115] Bi S., Wang H., Kuang W. (2018). Stem cell rejuvenation and the role of autophagy in age retardation by caloric restriction: An update. Mech. Ageing Dev..

[116] Katewa S.D., Akagi K., Bose N., Rakshit K., Camarella T., Zheng X., Hall D., Davis S., Nelson C.S., Brem R.B. (2016). Peripheral Circadian Clocks Mediate Dietary Restriction-Dependent Changes in Lifespan and Fat Metabolism in Drosophila. Cell Metab..

[117] Challet E., Solberg L.C., Turek F.W. (1998). Entrainment in calorie-restricted mice: Conflicting zeitgebers and free-running conditions. Am. J. Physiol..

[118] Mendoza J., Graff C., Dardente H., Pevet P., Challet E. (2005). Feeding cues alter clock gene oscillations and photic responses in the suprachiasmatic nuclei of mice exposed to a light/dark cycle. J. Neurosci..

[119] Patel S.A., Velingkaar N., Makwana K., Chaudhari A., Kondratov R. (2016). Calorie restriction regulates circadian clock gene expression through BMAL1 dependent and independent mechanisms. Sci. Rep..

[120] Pivovarova O., Gogebakan O., Sucher S., Groth J., Murahovschi V., Kessler K., Osterhoff M., Rudovich N., Kramer A., Pfeiffer A.F. (2016). Regulation of the clock gene expression in human adipose tissue by weight loss. Int. J. Obes..

